# Optimal isolation of extracellular vesicles from pleural fluid and profiling of their microRNA cargo

**DOI:** 10.1002/jex2.119

**Published:** 2023-10-16

**Authors:** Tian Mun Chee, Hannah E. O'Farrell, Luize G. Lima, Andreas Möller, Kwun M. Fong, Ian A. Yang, Rayleen V. Bowman

**Affiliations:** ^1^ The University of Queensland Thoracic Research Centre The Prince Charles Hospital Chermside Queensland Australia; ^2^ Tumour Microenvironment Laboratory QIMR Berghofer Medical Research Institute Herston Queensland Australia; ^3^ Department of Otorhinolaryngology Chinese University of Hong Kong Shatin Hong Kong; ^4^ Li Ka Shing Institute of Health Sciences Chinese University of Hong Kong Hong Kong China

**Keywords:** exosomes, extracellular vesicles (EVs), microRNA (miRNAs), microvesicles, nanostring, pleural fluid

## Abstract

Pleural effusion occurs in both benign and malignant pleural disease. In malignant pleural effusions, the diagnostic accuracy and sensitivity of pleural fluid cytology is less than perfect, particularly for the diagnosis of malignant pleural mesothelioma, but also in some cases for the diagnosis of metastatic pleural malignancy with primary cancer in the lung, breast or other sites. Extracellular vesicles (EVs) carry an enriched cargo of microRNAs (miRNAs) which are selectively packaged and differentially expressed in pleural disease states. To investigate the diagnostic potential of miRNA cargo in pleural fluid extracellular vesicles (PFEVs), we evaluated methods for isolating the extracellular vesicle (EV) fraction including combinations of ultracentrifugation, size‐exclusion chromatography (SEC) and ultrafiltration (10 kDa filter unit). PFEVs were characterized by total and EV–associated protein, nanoparticle tracking analysis and visualisation by transmission electron microscopy. miRNA expression was analyzed by Nanostring nCounter® in separate EV fractions isolated from pleural fluid with or without additional RNA purification by ultrafiltration (3 kDa filter unit). Optimal PFEV yield, purity and miRNA expression were observed when PFEV were isolated from a larger volume of pleural fluid processed through combined ultracentrifugation and SEC techniques. Purification of total RNA by ultrafiltration further enhanced the detectability of PFEV miRNAs. This study demonstrates the feasibility of isolating PFEVs, and the potential to examine PFEV miRNA cargo using Nanostring technology to discover disease biomarkers.

## 1 INTRODUCTION

The pleural space normally contains 0.1–0.2 mL/kg of pleural fluid, with an influx of 0.5 mL per hour in healthy adults (Hunter & Regunath, 2023). Pleural effusion is characterized by accumulation of fluid within the pleural space bounded by visceral pleura applied to lung, and parietal pleura applied to the inner aspect of the thoracic wall. In adults, pleural effusion may be the initial manifestation of benign or malignant conditions. Most large unilateral pleural effusions in adults are due to malignancy, even in the absence of previously known cancer. Tumours causing pleural effusion may arise from pleura itself such as malignant pleural mesothelioma (MPM), or they may involve pleura by direct extension from adjacent lung, or metastasise haematogenously to pleura from remote sites such as breast or ovary (Jany & Welte, 2019).

The most useful initial diagnostic test for malignant effusion is cytological examination of the pelleted cellular fraction of pleural fluid obtained by thoracentesis (needle aspiration or catheter drainage) with immunohistochemical characterisation (Shivakumarswamy et al., 2012). Although pleural fluid cytology is a critical step in differentiating benign from malignant effusions, the cytological diagnosis can be consistently benign in effusions that subsequently prove to be malignant. Pleural fluid cytology may be non‐diagnostic in up to 70% of malignant effusions, and these cases will often require a more invasive type of biopsy for definitive diagnosis (Loveland et al., 2018). A recent meta‐analysis evaluating 6057 patients from 36 studies concluded that pleural fluid cytology has a diagnostic sensitivity of 58.2% in malignant pleural effusion, with high sensitivity for lung adenocarcinoma metastatic to pleura (83.6%) but unsatisfactory sensitivity for MPM (28.9%) (Kassirian et al., 2023).

Extracellular vesicles (EVs) are particles enclosed by a lipid bilayer without a functional nucleus (Théry et al., 2018), which are naturally released from all types of cells. While they are conventionally characterised by size as apoptotic bodies (>1000 nm), microvesicles (100–1000 nm) and exosomes (30–150 nm), these size ranges remain controversial (Akers et al., 2013; Jurj et al., 2020; O'Farrell & Yang, 2019). Each type of extracellular vesicle (EV) arises from distinct biogenesis pathways: (1) apoptotic bodies—programmed cell death; (2) microvesicles—outward budding of the plasma membrane through exocytosis; and (3) exosomes—endocytosis originating from early endosomes, processed through maturation of multivesicular bodies, and fused with lysosomes or plasma membrane before being released into the extracellular space (Jurj et al., 2020; O'Farrell & Yang, 2019). EVs are postulated to maintain cellular homeostasis by removing biomolecules produced in excess, and by transferring molecular cargo to recipient cells to facilitate intercellular communication. They also exert pathological effects by transferring oncoproteins, oncomirs, and non‐coding RNAs, thus imposing unwarranted gene regulation on recipient cells at post‐transcriptional level (Kalluri & LeBleu, 2020; Mohammadi et al., 2020; Nawaz et al., 2016; Valadi et al., 2007). MicroRNA (miRNA) packaged within EV cargo can participate in either tumorigenesis or cell growth inhibition (Boukouris & Mathivanan, 2015; Hao et al., 2007; Keller et al., 2009). In tumours, the composition of EV cargo falls under the influence of genomic and genetic alterations, and EV–derived miRNAs or proteins can play crucial roles in tumour growth, apoptosis and cancer metastasis (Lou et al., 2016).

The diagnostic potential of miRNAs in pleural effusions has been demonstrated in multiple studies (Birnie et al., 2019; Cappellesso et al., 2016; Girolami et al., 2022; Sorolla et al., 2021; Zhang et al., 2021). In pleural fluids, miR‐21 and miR‐126 showed a sensitivity of 0.86 and a specificity of 0.87 in distinguishing between malignant mesothelioma and reactive mesothelial cells (Cappellesso et al., 2016). In the cellular fraction of pleural fluid, a three‐miRNA signature (miR‐143, miR‐210 and miR‐200c) achieved an area under the curve (AUC) of 0.9887 in distinguishing between MPM and lung adenocarcinoma (Birnie et al., 2019). In another study, miR‐130a was reported to differentiate malignant mesothelioma from lung adenocarcinoma at sensitivity of 0.77 and specificity of 0.67 (Cappellesso et al., 2017). In lung cancer, independent studies have demonstrated that EV‐derived miRNA signatures can distinguish between patients with early‐stage lung cancer and healthy controls (Lu et al., 2018; Wang et al., 2015).

Standard cytological processing of pleural fluid involves centrifugation of cells and other sediments at 600 *x g* (10 minutes) for cytospins, smears and cell block preparations (Engels et al., 2019; Saqi, 2016). Because the centrifugation speed required to isolate EVs, particularly microvesicles and exosomes (≥ 20,000 *x g*), is much higher than that required for pellet cells (Théry et al., 2018), the EV component of pleural fluid remains in the supernatant fraction during standard processing of clinical thoracentesis samples. Thus, EV content is not examined in routine diagnosis.

Although EVs in malignant pleural effusions are potentially enriched with tumour markers that could be diagnostically useful, currently no standardised method has been identified to isolate pleural fluid extracellular vesicles (PFEV) for this purpose. We therefore assessed the yield and purity of PFEV isolated from pleural fluid by various techniques and examined the effect of different EV isolation methods on the quality of miRNA expression profiles obtained from Nanostring nCounter® miRNA expression assays. This proof–of–principal study demonstrates the feasibility of interrogating PFEV miRNA cargo in pleural disease states by using isolation methods optimised for purpose, and supports future exploration of the diagnostic potential of EV cargo in pleural effusions.

## 2 MATERIALS AND METHODS

### 2.1 Ethics approval

Patients who underwent thoracentesis at The Prince Charles Hospital gave written informed consent to donate pleural fluid remaining after diagnostic pathology examination. This study was approved by Metro North Hospital and Health Service (MNHHS) Human Research Ethics Committee (HREC/18/QPCH/312).

### 2.2 Cell‐free pleural fluid preparation (CFPF)

Archival pleural fluid was centrifuged at 600 *x g* for 7 min to separate cells and supernatant. The supernatants were stored at −80 ⁰C for up to 10 years, then thawed at room temperature and sieved through a 40 μm cell strainer (DKSH, Cat. No. 15‐1040‐1) to remove remaining tissue fragments, blood clot and large particles. The filtrate was then centrifuged at 800 *x g* for 10 min at 4⁰C. After removing visible cell pellet, the resulting supernatant (referred to as CFPF) was used for PFEV isolation.

### 2.3 Pleural fluid extracellular vesicle isolation

#### 2.3.1 Ultracentrifugation

Ultracentrifugation was performed at 100,000 *x g* for 1 h and 40 min at 4°C (*w^2^t* = 5.46e10) using a Beckman Optima XPN‐100 ultracentrifuge, and 50.2 Ti rotor (Beckman Coulter, IN).

#### 2.3.2 Size‐exclusion chromatography (SEC)

SEC was performed using a qEV Original/70 nm Legacy Column and Automatic Fraction Collector (Izon Science, Cat. No. SP1) according to manufacturer's instruction. 0.5 mL sample fluid was processed, yielding a 1.5 mL eluate.

#### 2.3.3 Ultrafiltration (in PFEV isolation)

Eluate collected from SEC was loaded onto a 10 kDa Amicon Ultra‐4 Centrifugal Filter Unit (Merck, Cat. No. UFC801024) and the column was centrifuged at 4,000 *x g* at 4°C for 5 or 10 min until the eluate was concentrated to a 0.15 mL volume.

### 2.4 PFEV isolation processes generating preparations A—H

**FIGURE 1:**
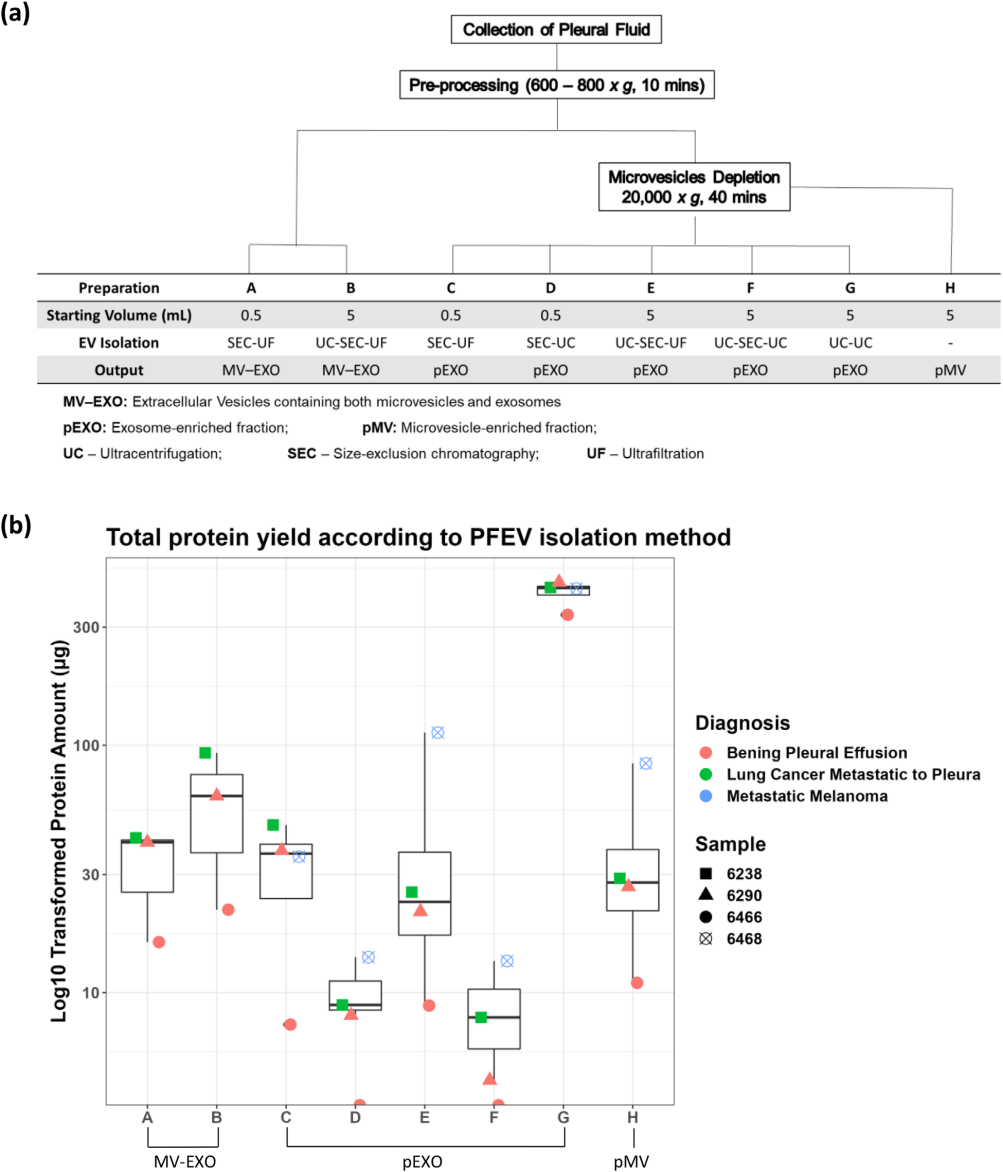
Method matrices for isolating pleural fluid extracellular vesicles from cell‐free pleural fluids. **(a**) Pleural fluid was spun at 600–800 *x g*, resulting in cell‐free pleural fluid (CFPF). CFPF was processed to generate MV–EXO, pMV and pEXO fractions (Preparations A—H). The table lists input volumes of pleural fluid used for eight different EV preparations employing ultracentrifugation, size‐exclusion chromatography and/or ultrafiltration steps. **(b)** Total protein yield (μg) in EV fractions prepared from four pleural fluid samples (cases PF6238, PF6290, PF6466 and PF6468) using eight different methods for EV isolation.

PFEV isolation methods combining ultracentrifugation, SEC and/or ultrafiltration were optimised using pleural fluid samples obtained from four different donors (PF6238, PF6290, PF6466, and PF6468). Final EV products were adjusted to 0.15 mL with 1X PBS prior to protein quantitation and expression analyses. Figure 1a summarises CFPF starting volumes and processes used for each method of PFEV isolation.

#### 2.4.1 Fractions containing both microvesicles and exosomes (MV–EXO; Preparations A and B)

A total of 5.5 mL CFPF from each case (PF6238, PF6290 and PF6466) was used to generate preparations A and B. **Preparation A**: 0.5 mL CFPF was subjected to SEC, followed by ultrafiltration. **Preparation B**: 5 mL CFPF underwent ultracentrifugation. The pellet was resuspended in 0.5 mL of 1X PBS, and then processed by SEC and ultrafiltration.

#### 2.4.2 Putative microvesicle‐enriched fraction (pMV; Preparation H)

20 mL CFPF was centrifuged at 20,000 *x g* for 40 min, resulting in pMV. The pellet was resuspended in 300 μL of 1X PBS for protein analysis. The remaining pMV‐depleted CFPF was used to generate putative exosome‐enriched fractions.

#### 2.4.3 Putative exosome‐enriched fractions (pEXO; Preparations C—G)

1 mL pMV‐depleted CFPF was divided into two aliquots of 0.5 mL, each subjected to SEC. The eluates were concentrated by either ultrafiltration (**Preparation C**) or ultracentrifugation (**Preparation D**).

15 mL pMV‐depleted CFPF was processed by ultracentrifugation. The resulting pellet was resuspended in 1.5 mL 1X PBS, which was then subdivided into three aliquots. 1 mL of EV resuspension was processed separately by SEC (0.5 mL at a time), with each eluate further concentrated by either ultrafiltration (**Preparation E**) or by ultracentrifugation (**Preparation F**). The remaining 0.5 mL EV resuspension was first diluted to 2 mL with 1X PBS, then further concentrated by ultracentrifugation (**Preparation G**).

### 2.5 Protein quantitation

Micro BCA Assay (ThermoFisher, Cat. No. 23235) was used to quantitate total protein in PFEV fractions. Bovine serum albumin standard was prepared at concentrations of 0, 0.25, 0.5, 5, 25, 50, 100, 200 μg/mL to generate a standard curve. 150 μL sample volume prepared in the presence of 10% RIPA buffer (ThermoFisher, Cat. No. 89900) supplemented with 1X Halt™ protease inhibitor cocktail (ThermoFisher, Cat. No. 87785) was incubated with 150 μL of working buffer for 2 h at 37⁰C according to manufacturer's instructions. Absorbance was measured at 562 nm on a FLUOstar Omega plate reader (BMG Labtech). GraphPad Prism 8 software version 8.0.1 (San Diego, California) was then used to interpolate readouts against the standard curve. Assays were performed in duplicate, and the mean of duplicates was plotted for the estimation of total protein yield.

### 2.6 Western blotting

Western blotting was performed with iBlot 2 Dry Blotting System (ThermoFisher Scientific) following manufacturer's instructions. 2 μg protein sample was prepared with 10 μL Bolt™ LDS Sample Buffer 4X (ThermoFisher Scientific, Cat. No. B0007) and 4 μL Bolt Reducing Agent 10X (ThermoFisher Scientific, Cat. No. B0009). The mixture was heated at 70 ⁰C for 10 min and loaded for gel electrophoresis (100 V for 90 min). Protein was transferred onto the PVDF membrane (ThermoFisher Scientifix, Cat. No. IB24002) using an iBlot 2 Gel Transfer Device at 25 V for 6 min. The blot was blocked for 30 min in Tris‐Buffered Saline supplemented with 0.1% Tween (TBS/0.1% Tween) and 5% skim milk powder. Novex™ Sharp Pre‐Stained Protein Standard (Thermo Fisher Scientific, Cat. No. LC5800) was used in all blots. Primary antibody incubation was performed overnight as follows: Anti‐Albumin (1:1000, Cell Signalling Technology, Cat. No. 4929S), Anti‐FLOT1 (D2V7J) XP® Rabbit mAb (1:1000; Cell Signalling Technology, Cat. No. 18634S) and Anti‐CD9 (D8O1A) Rabbit mAb (1:5000, Cell Signalling Technology, Cat. No. 13174S). The blot was then incubated in Goat anti‐Rabbit IgG (H+L) Secondary Antibody, HRP (1:10000, ThermoFisher, Cat. No. 31460) for 1 h. TBS/0.1% Tween was used to wash the blots between blocking and antibody incubations. Finally, the blots were developed using SuperSignal™ West Femto Maximum Sensitivity Substrate (ThermoFisher, Cat. No. 34094) for 5 min and images were detected by the ImageQuant™ LAS 4000 system (GE Healthcare).

### 2.7 Nanoparticle tracking analysis (NTA)

Two PFEV samples from PF6219 were analyzed using the NanoSight NS300 instrument (Malvern Instruments, Amesbury, UK) equipped with a blue 488 nm laser, a high sensitivity sCMOS camera, and a syringe pump. The samples were diluted and filtered with 1X PBS using a 0.4 μm Minisart filter (Sartorius, Germany), loaded into a 1 mL syringe connected to a syringe pump and processed at a setting of 50 with an automatic temperature setting of 22°C to obtain 40−100 particles/view. Ten videos were recorded using camera level 10–11 at 1‐min intervals. Data was analyzed using NanoSight NTA software (version 3.2; Malvern Panalytical Ltd.) with the detection threshold set at 4–5 and screen gain at 10–11 to track the greatest number of particles with minimal background. Data from ten recording time frames along with averaged data were plotted using NTA software.

### 2.8 Transmission electron microscopy (TEM)

PFEV visualisation by TEM was performed at UQ Centre for Microscopy and Microanalysis (UQCMM). Negative staining was performed on undiluted PFEV on Formvar filmed, carbon‐coated and flow‐discharged EM Grids (ProSciTech). Images were acquired with a JEOL JEM‐1011 Transmission Electron Microscope operated at 100 kV equipped with a SIS Morada Camera, and acquisition software Olympus iTEM 5.2 with scale bars of 2 μm, 1 μm and 0.5 μm (500 nm).

### 2.9 Total RNA extraction

Total RNA from PFEV was extracted using QIAzol reagent (Qiagen, Cat. No. 79306) and Norgen RNA Clean‐Up and Concentration Micro‐Elute Kit (Norgen Biotek, Cat. No. 61000) following protocol B (96–100% Ethanol in Step 1b) and manufacturer's instructions. Briefly, PFEV pellets (from the final ultracentrifugation) were each resuspended in 0.5–0.7 mL of QIAzol reagent. The mixture was homogenised using a vortex mixer and incubated at room temperature for 5 min. 140 μL of chloroform was added to the mixture, incubated at room temperature for 3 min and centrifuged at 12,000 *x g* for 15 min at 4⁰C. After separation of aqueous and organic phases, the upper aqueous phase containing RNA was collected into a new RNase‐free microtube and 100% Ethanol was added. Total RNA of PFEV was eluted in 10 μL of elution buffer.

#### 2.9.1 RNA purification by ultrafiltration

400 μL of UltraPure™ DNase/RNase‐free Distilled Water (ThermoFisher, Cat. No. 10977015) was added to the extracted total RNA, and the mixture was centrifuged at 14,000 *x g* for 90 min in Amicon Ultra‐0.5 mL 3 kDa Centrifugal Filter (Merck, Cat. No. UFC5000324). The column was then inverted into a fresh microtube and centrifuged at 8,000 *x g* for 2 min to collect purified total RNA.

### 2.10 Nanostring miRNA expression assay

The extracted total RNA (including miRNA) from PFEV was quantified using Qubit™ 4 Fluoromater (ThermoFisher Scientific, Cat. No. Q33226) with Qubit™ High Sensitivity RNA (ThermoFisher Scientific, Cat. No. Q32852), followed by RNA quality assessment using the Agilent 2100 Bioanalyzer with Small RNA assay (Agilent Technologies, Inc., Cat. No. 5067–1548) prior to miRNA expression assay. The Nanostring nCounter® human miRNA expression assay panel (NanoString Technologies, Inc., Cat. No. 150629) screens up to 827 miRNAs (in 798 miRNA probes) that are highly curated in miRBase 22 (released on 12^th^ March 2018). The assay was performed following nCounter miRNA Expression Assay user manual (MAN‐C0009‐07; v. April 2018). Hybridisation assay for tagged miRNAs and capture/reporter probes with colour‐coded sequence specific to each miRNA target was incubated for at least 12 h (maximum of 17 h). Post‐hybridisation samples were then loaded onto a SPRINT cartridge (NanoString Technologies, Inc., Cat. No. 100078) and processed on the Nanostring nCounter® SPRINT Profiler. Data was acquired through instrument‐automated fluorescence microscope scanning with direct counts of target‐probe complexes, which was then processed by Nanostring nSolver™ software Version 4.0.70 (NanoString Technologies, Inc.).

### 2.11 Statistical analysis

Nanostring nSolver™ software was used to process raw expression data generated from PFEV total RNA. Unsupervised cluster analysis was performed using ‘pheatmap’ package built under R version 4.2.3 with RStudio (Build 554) to show raw count distribution of PFEV miRNA expression.

## 3 RESULTS

### 3.1 Subjects

Eleven cases of pleural fluid used in this study had clinical diagnoses as follows: MPM (*n* = 3), metastatic melanoma (*n* = 1), lung cancer metastatic to the pleura (*n* = 3), and benign effusions (*n* = 4) (Table 1). The clinical diagnosis of malignant effusions was confirmed by cytology or histopathology. The benign cases had no evidence of malignancy on pleural fluid cytology, and confirmation of the final diagnosis was based on clinical and imaging follow‐up, confirming that they were not subsequently diagnosed with malignancy.

**TABLE 1:** Clinical diagnosis of pleural fluid subjects.

Subject	Clinical diagnosis	Sex	Age (at specimen collection)
PF6066	Malignant Pleural Mesothelioma	Female	71
PF6129	Malignant Pleural Mesothelioma	Female	87
PF6238	Lung cancer metastatic to pleura	Male	57
PF6246	Benign Pleural Effusion	Male	81
PF6290	Benign Pleural Effusion	Male	84
PF6466	Benign Pleural Effusion	Male	97
PF6468	Metastatic melanoma	Male	84
PF7045	Lung cancer metastatic to pleura	Female	52
PF7278	Malignant Pleural Mesothelioma	Female	72
PF7432	Lung cancer metastatic to pleura	Male	81
PF21153	Benign Pleural Effusion	Male	65

### 3.2 EV isolation by various method matrices demonstrated consistent protein outputs

The total protein yield from various PFEV isolation methods were compared in four pleural fluid samples. The total amount of protein (μg) yielded from each preparation method (**Preparation A—**H, Figure 1a) was presented in Figure 1b. The volume of CFPF used in PFEV isolation did not determine protein yield. In fact, the amount of protein obtained from 0.5 mL CFPF‐equivalent input was greater than that from 5 mL (**Preparation C** vs. **E; D** vs. **F**). Protein yield was higher when the final PFEV sample was concentrated by ultrafiltration than when processed through ultracentrifugation (**Preparations C** vs. **D**; **E** vs. **F**). Among the pEXO fractions (**Preparation C—G**), **Preparation G** (prepared by consecutive ultracentrifugation steps) yielded the highest amount of protein.

The protein yield of pMV fractions (**Preparation H**) varied considerably between samples, ranging from 43.78ug to 337.82 μg isolated from 20 mL CFPF. The protein amount presented in Figure 1 was normalised to 5 mL CFPF input volume. For three of four samples, higher total protein yields were observed in fractions putatively containing both microvesicles and exosomes (MV‐EXO) than in the corresponding exosome‐enriched fractions (pEXO) (comparison of Preparations **B** and **E**, using 5 mL CFPF for EV isolation. MV‐EXO fractions isolated from an initial sample volume of 5 mL CFPF (**Preparation B**) yielded more protein than from 0.5 mL initial sample volume (**Preparation A**).

### 3.3 Discordance between total protein yield and expression of EV‐associated protein markers

**FIGURE 2:**
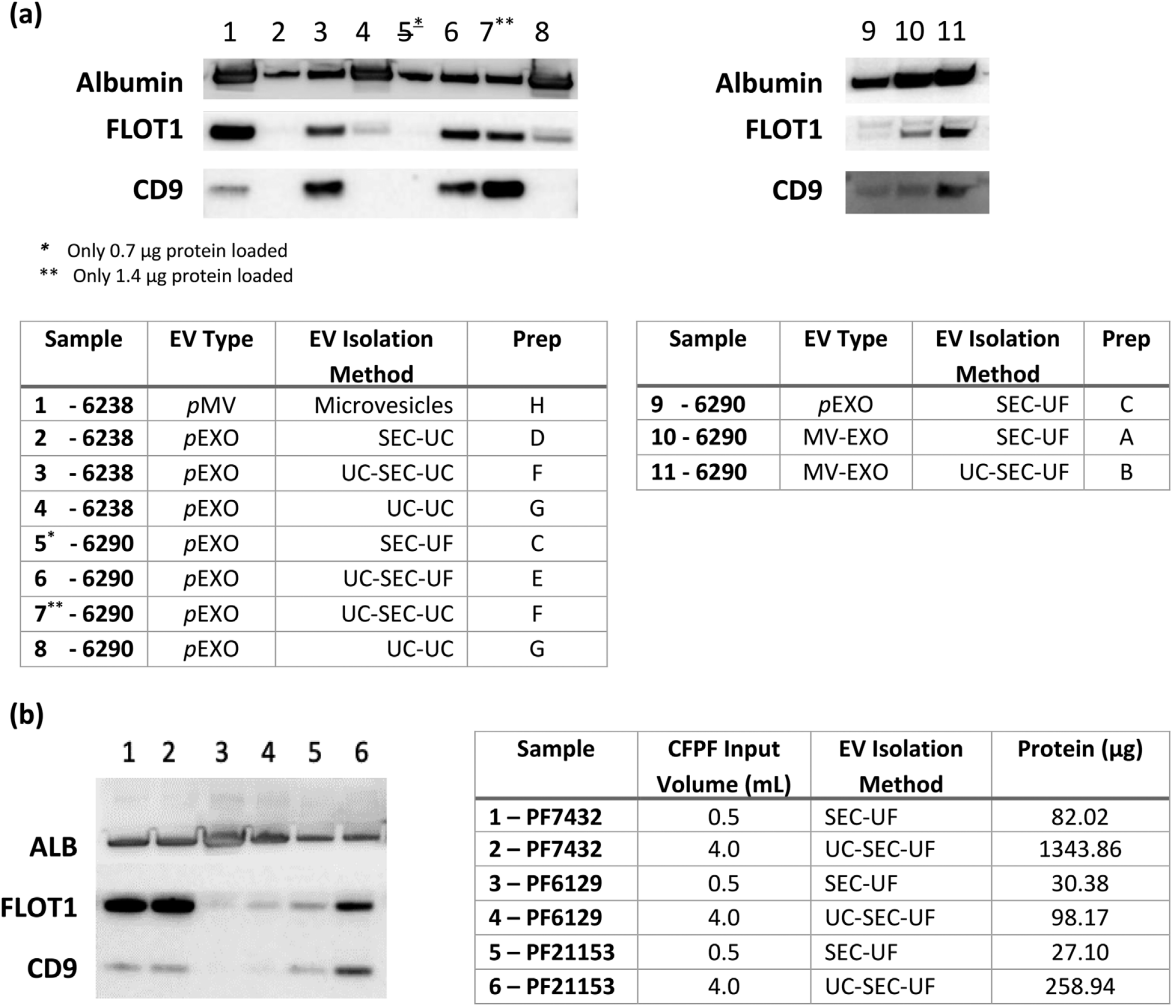
Western blotting for comparison of PFEV isolation preparations. **(a)** The expression of Albumin, FLOT1 and CD9 in PFEV recovered from **Preparations A—H** in samples PF6238 and PF6290. 2 μg protein was loaded per lane, except for lane 5 (0.7 μg –due to technical error) and lane 7 (1.4 μg—insufficient sample). **(b)** Expression of Albumin, FLOT1 and CD9 in PFEV of PF7432 (Lanes 1, 2), PF6129 (Lanes 3, 4) and PF 21153 (lanes 5, 6). All lanes were loaded with 2ug total protein. Lanes 1, 3, and 5 represent PFEV prepared by SEC–UF from 0.5 mL CFPF, while lanes 2, 4, and 6 were prepared by UC–SEC–UF from 4 ml CFPF. *Table on the right*: The input volume of cell‐free pleural fluid for PFEV isolation and the resulting protein yield were presented.

Regardless of preparation method, the total protein yield was not associated with PFEV abundance as assessed by the expression of EV–specific protein markers. In Figure 2a, PFEV isolated from 5 mL of CFPF processed through UC‐SEC demonstrated high FLOT1 and CD9 despite low total protein yield (lanes 3, 6, 7 and 11), whereas PFEV isolated from 0.5 mL of CFPF (with relatively high total protein yield) showed low expression of FLOT1 and CD9 (lanes 2, 9, 10). Similarly, even though isolation of PFEV through two rounds of ultracentrifugation produced the highest total protein yield among pEXO fractions, there was low expression of FLOT1 expression and no CD9 in these samples (lanes 4 and 8).

EV‐specific markers were demonstrated most clearly on western blots when the EV isolation method included SEC. Figure 2a shows that pEXO fractions isolated with SEC demonstrated lower levels of Albumin (lanes 2,3,6,7) than preparations without SEC (lanes 4 and 8), indicating that SEC may improve EV purity. Additionally, EV‐specific markers were higher in PFEVs retaining microvesicles: Figure 2a, lane 1 shows high expression of FLOT1 in the pMV fraction of PF6238 indicating abundant EVs, and the MV–EXO fraction of PF6290 with microvesicle retention (lanes 10 and 11) demonstrated greater FLOT1 and CD9 expression than the corresponding pEXO fraction (lane 9). However, the MV–EXO fractions also expressed higher levels of Albumin, indicating greater co–isolation of protein not originating from EVs.

To further optimise EV yield and purity, MV–EXO fractions were examined in three independent samples (PF7432, PF6129, and PF21153). Two different input volumes for PFEV isolation were tested: (1) SEC–UF: 0.5 mL was the default input volume for qEV Original Legacy column; (2) UC–SEC–UF: a higher input volume of 4 mL pleural fluid was first concentrated down to a 0.5 mL input volume for SEC (Figure 2b). PF7432 was highly EV abundant (Lanes 1 and 2 demonstrating strong expression of EV–associated proteins, FLOT1 and CD9, regardless of initial volume or preparatory method), whereas PF6129 demonstrated low EV abundance (lanes 3, 4). In sample 21153, the expression of FLOT1 and CD9 was stronger in PFEV isolated from 4 mL CFPF by UC–SEC–UF (lane 6) than in EVs derived from 0.5 mL volume by SEC–UF (lane 5), demonstrating the advantage of maximizing EV recovery by concentrating a higher input volume for PFEV isolation using ultracentrifugation. However due to inter‐patient variability in EV–associated protein expression, a conclusion could not be drawn based on the results from PF21153 alone.

### 3.4 Sensitive PFEV quantitation by NTA

**FIGURE 3:**
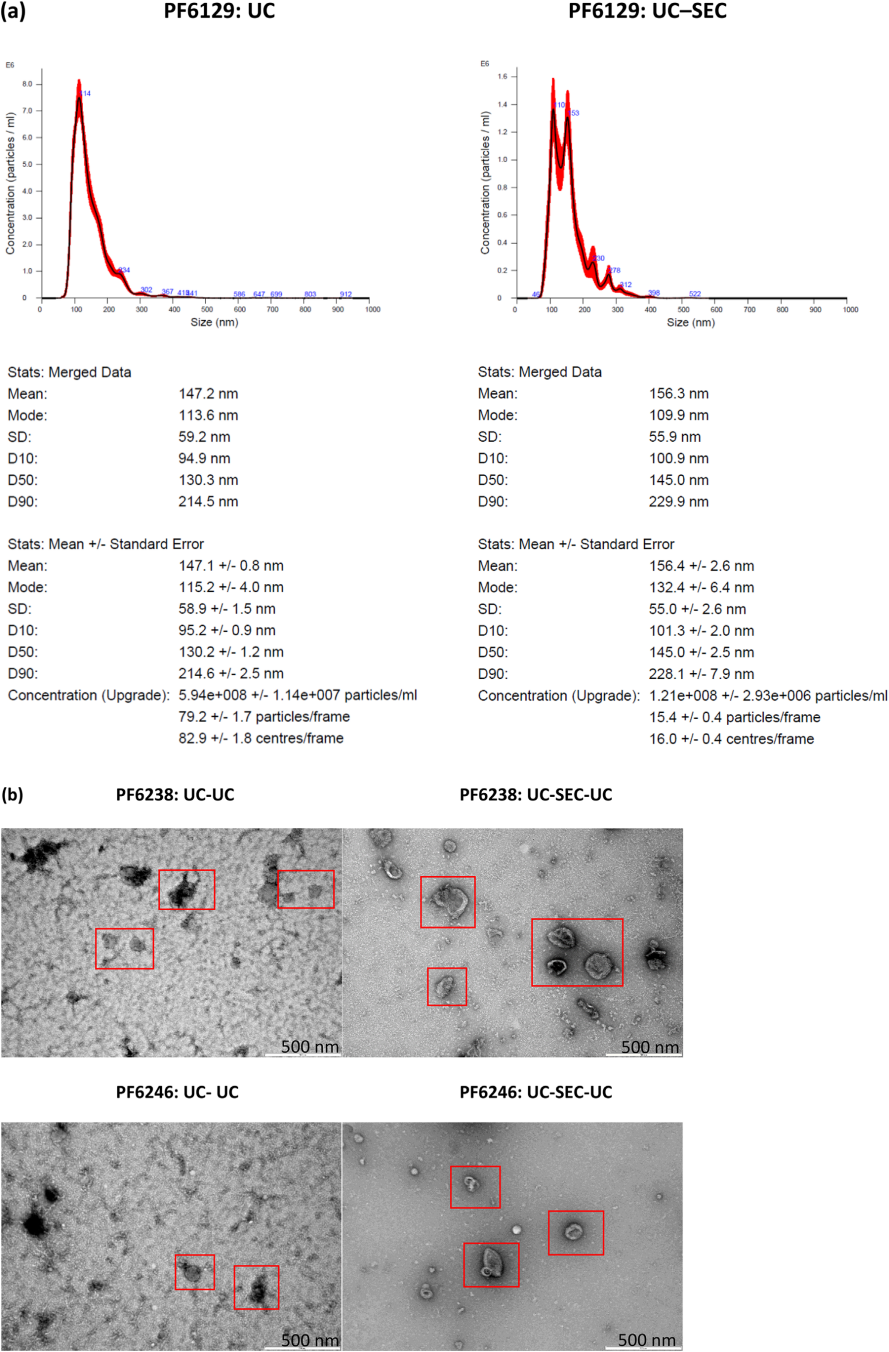
PFEV characterisation by nanoparticle tracking analysis and transmission electron microscopy. **(a) NTA**: PFEV of PF6129 isolated by UC alone showed 5.94 × 10^8^ ± 1.14 × 10^7^ particles per mL final EV volume, whereas UC‐SEC yielded 1.21 × 10^8^ ± 2.93 × 10^6^ particles per mL. The 10^th^, 50^th^ and 90^th^ percentiles of the averaged EV particle sizes were 94.9 nm, 130.3 nm and 214.5 nm for UC, whereas 100.9 nm, 145.0 nm and 229.9 nm for UC‐SEC. **(b) TEM**: PFEV of PF6238 and PF6246 isolated by UC–UC or UC–SEC–UC preparation were evident as indicated within the red boxes. Images of UC–SEC–UC derived PFEVs show well‐defined cup‐shaped particles of varying sizes, with lower background in comparison with corresponding samples isolated by UC–UC.

The low level of expression on western blots of EV–associated markers FLOT1 and CD9 in PF6129 suggested low EV abundance in this sample, so NTA was used to determine whether EVs were in fact present in the PF6129 EV fraction. PFEV prepared by either UC or UC‐SEC from 2 mL of CFPF showed particles in the expected exosome and microvesicle size range of 50–400 nm. Particle sizes ranged above 400 nm were not detected from NTA due to the use of 0.4 μm Minisart filter during sample preparation for NTA. When isolated by ultracentrifugation alone, the relative particle concentration was 5.94 × 10^8^ ± 1.14 × 10^7^ particles per mL (with a size peak at 114 nm); whereas when PFEV were isolated by UC–SEC, there were fewer particles per mL (1.21 × 10^8^ ± 2.93 × 10^6^), and two size peaks were observed at ∼110 nm and ∼150 nm (Figure 3a). Thus, NTA verified the presence of EVs in PF6129 despite low levels of FLOT1 and CD9 expression.

### 3.5 Visualisation of EVs by TEM

PFEV from two pleural fluid samples (PF6238 and an independent case, PF6246) isolated by either UC–UC or UC–SEC –UC were examined by TEM. At a resolution of 500 nm, TEM images confirmed the presence of EVs with cup‐shaped morphology in both samples prepared by either method (Figure 3b), although high background was observed in UC–UC preparations.

### 3.6 Effects of initial sample volume and total RNA extraction methodology on miRNA detectability in PFEV

To optimise miRNA detection, different EV preparation methods with or without RNA purification (RNAPur; by ultrafiltration) were applied to sample PF7278 as follows: **a)** UC–UC^2.5^; **b)** UC–UC, RNAPur^2.5^; **c)** UC–SEC–UC^2.5^; **d)** UC–SEC–UC, RNAPur^2.5^; and **e)** UC–SEC–UC, RNAPur^5^, where **^2.5^** refers to input volume of 2.5 mL, and **^5^** refers to input volume of 5 mL. Raw expression data was presented in a heatmap with Log_10_ transformed raw count value (Figure 4b). 523 miRNA probes with no expression in any PF7278 preparations were excluded, leaving only 275 miRNA probes represented in the heatmap. The application of SEC and RNAPur effectively improved miRNA detectability, whereby higher expression of miRNAs was observed (**samples b**, **c**, **d** and **e**, compared to **sample a**). Further, the expression for most miRNAs in **sample e** was higher than in **sample d**, in which CFPF input volume was doubled in PFEV isolation. Figure 4a shows the fold‐change in miRNA raw count values between **sample d** and **e**, miRNA values < 50 in both samples were removed from the heatmap. Except for four miRNAs that had expression levels lower in **sample e** than in **d** (0.8‐ to 0.9‐fold decrease), the expression of 143 miRNAs were higher in **sample e** than in **d** (1.1‐ to 3.5‐fold increase).

FIGURE 4Heatmap of miRNA expression in PF7278; and PF6066, PF6238, PF6246 and PF7045. **(a)** The fold change in miRNA raw count values between sample d and e was presented. **(b)** 275 miRNAs were presented at Log_10_ transformed raw count expression value. Higher expression of most miRNAs was observed with the application of SEC, RNA purification, more significantly by increasing the volume of CFPF used in PFEV isolation. **(c)** Heatmap of miRNA expression at log_10_ transformed raw count expression value. PFEV of four pleural fluid cases (PF6066, PF6238, PF6246, PF7045) were isolated by Method 1 (UC–qEV–UC, RNAPur from 5 mL CFPF input volume) or Method 2 (UC–UC from 16 mL CFPF input volume). The number of miRNAs with raw count values < 50 or ≥ 50 was presented.

**Figure:**
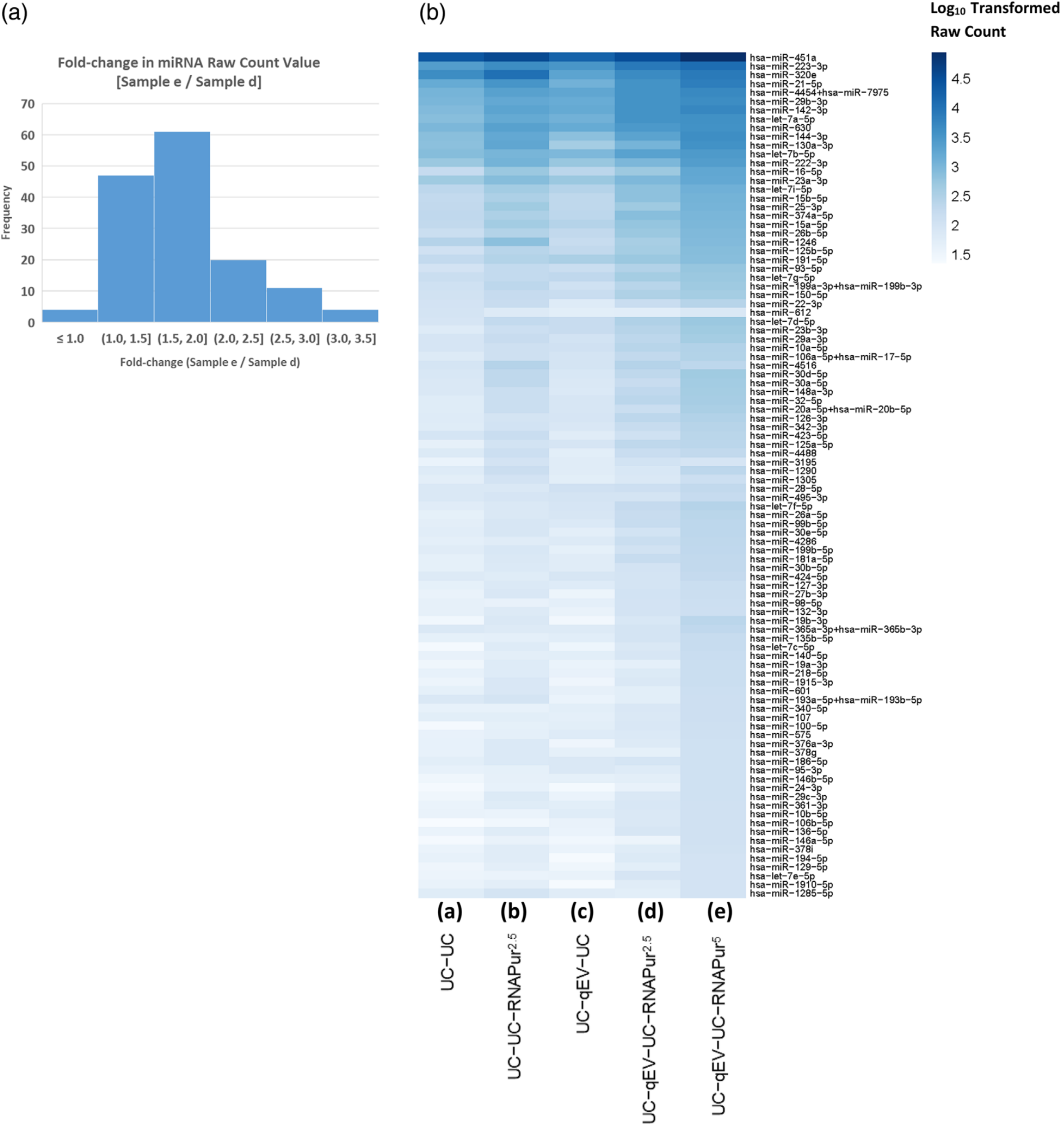

**Figure:**
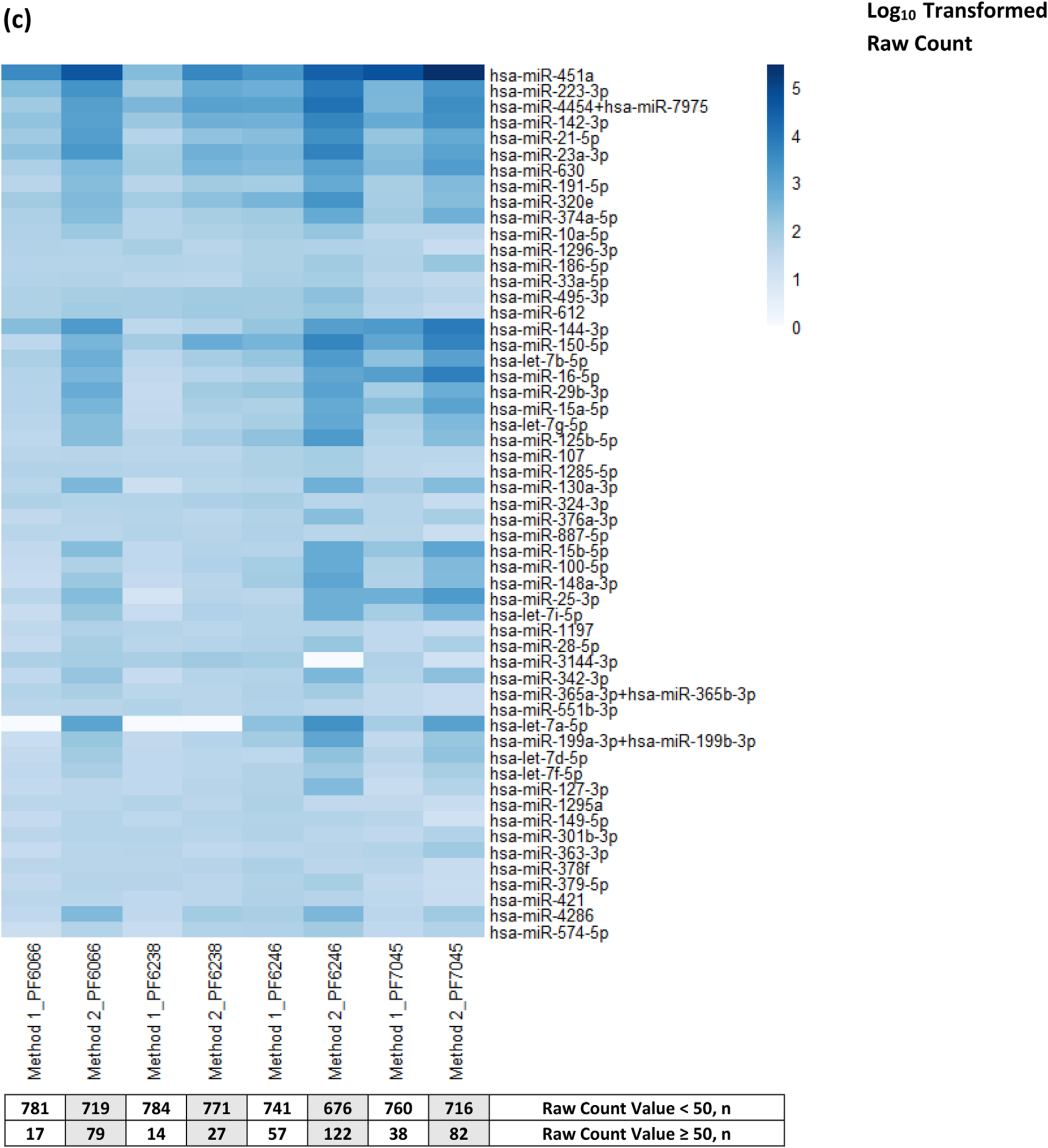

The miRNA expression for an independent sample set (PF6066, PF6238, PF6246, PF7045) isolated by two different methods was presented in Figure 4c. Pragmatically, Method 1 (UC–SEC–UC, RNAPur from 5 mL CFPF input volume; same as the method used for **sample e**) was validated further in these four samples. Method 2 (UC—UC from 16 mL CFPF input volume) was included as another approach to reduce processing time and consumable costs but a much higher volume of CFPF was used to compensate for the elimination of SEC and RNA purification. The heatmap shows only those miRNAs that were expressed at ≥ 50 raw counts in at least one of the eight PFEV samples tested in this experiment. Both methods showed at least 14 miRNAs conferred a raw count value ≥ 50. Although SEC and RNA purification processes were eliminated in Method 2, all samples showed an overall greater miRNA expression than those processed by Method 1. However, a direct comparison of both methods would not be justified as Method 1 and Method 2 applied different methods and input volume for PFEV isolation. The results from this experiment showed that both Method 1 and Method 2 can be considered for PFEV isolation.

## 4 DISCUSSION

There is emerging evidence that EVs in biofluids contain nucleic acids with diagnostic potential in cancer, including for malignant pleural diseases (Liu et al., 2022; Möller & Lobb, 2020). Pleural diseases commonly present as pleural effusion but current diagnostic testing based on relatively non‐invasive pleural fluid sampling lacks diagnostic sensitivity for certain conditions (Kassirian et al., 2023; Loveland et al., 2018). miRNAs are circulated in body fluids, such as pleural fluid, plasma or urine, through various mechanisms—passive leakage in relation to various biological events such cell injury or inflammation, active secretion through membrane vesicles such as EV, or by formation of protein—miRNA complexes (Nik Mohamed Kamal & Shahidan, 2020). Thus, the miRNA cargo of effusion EVs is of increasing interest in the search for improved diagnostics for pleural diseases. As EVs can be isolated and enriched only through high‐speed ultracentrifugation, SEC, or other specialised techniques, the current repertoire of diagnostic tests does not include testing of the EV fraction of pleural fluid (Théry et al., 2018). A relatively pure EV fraction is required in order to analyse the actively packaged EV nucleic acid cargo in pleural effusions. Although multiple methods of isolating EVs from biofluids have been described, there is limited information about the optimal method of isolating the EV fraction of pleural fluid (Gheinani et al., 2018; Lobb et al., 2015).

We therefore tested methods of isolating EVs from pleural fluid using various sequential processing steps in combination, and examined relationships between overall protein yield, specific EV–associated protein expression, and EV purity. Then, we tested the applicability of the various isolation methods to the specific downstream purpose of analysing pleural fluid EV miRNA content. EV extraction methods were designed to generate separate putative microvesicle–retaining (ultracentrifugation at 20,000 *x g*) or exosome–enriched fractions (ultracentrifugation at 100,000 *x g* or SEC) (Lobb et al., 2015), and as might be expected we found that fractions retaining both microvesicles and exosomes demonstrated higher PFEV abundance.

We found that total protein yield did not correlate with expression of the EV–associated proteins, FLOT1 and CD9. In fact, PFEV isolation methods associated with greatest expression of EV positive markers had the lowest overall protein content. Ultracentrifugation is a *de facto* gold standard EV isolation method in widespread use due to its cost‐effectiveness (Li et al., 2017). With ultracentrifugation only isolation methods, EVs were detectable by NTA and TEM, but despite high total protein yield and high albumin expression with this method, the expression of EV‐ associated proteins such as FLOT1 was low. These findings could arise from co‐isolation of proteins that do not originate from EVs. By including SEC in the isolation method, we could achieve superior EV definition and lower background on TEM images, and the lower albumin expression was consistent with efficient depletion of co‐isolated non–EV components with application of SEC. In a nutshell, ultracentrifugation and SEC could be used as a single technique or in combination to achieve an optimal EV yield and purity, depending on the objective of the research. Ultracentrifugation offers a cost–effective solution to isolate EV but the EV purity is lower than SEC by qEV column. SEC by qEV column is a favourable approach if superior EV purity is required for downstream experiments, such next generation sequencing.

In relation to the specific downstream purpose of analysing EV miRNA cargo, we reported enhanced detectability of miRNAs by Nanostring in EVs isolated by methods including SEC and additional RNA purification manoeuvres. However, this could also be achieved by starting with a higher sample input volume, even omitting SEC and RNA purification steps with benefits of reduced cost and improved time efficiency. Nonetheless, SEC and RNA purification procedures could offer improved miRNA detectability for EV miRNA detection in other biofluids such as plasma, saliva, and exhaled breath condensate, where obtaining a sufficiently high volume sample from which to isolate EVs is often challenging.

Several shortcomings to this study are acknowledged. Firstly, the studies were performed on archival pleural fluids that were stored for up to ten years at −80°C. The expression of miRNA in pleural fluid could be influenced by factors due to natural causes (type of disease, stage and progression of disease), treatment course, inconsistency during sample handling, particularly processing time incurred from thoracentesis to storage of pleural fluid at ‐80°C (Calin et al., 2005; Cervena et al., 2021; Giza et al., 2014; Mayr et al., 2017). Long‐term storage and freeze–thaw cycles may alter miRNA expression packaged in PFEV. Therefore, the results from this study will require technical validation in freshly collected pleural fluids. Secondly, the pleural fluid samples available for testing were limited in both number and volume. As a result, full‐scale EV particle analysis (quantitative and visualisation) could not be executed on all samples. Some experiments were limited to very few samples restricting the number of experimental replications that were possible. PFEV concentrated by ultrafiltration using the 10 kDa Amicon Ultra‐4 Centrifugal Filter Unit was only tested for the expression of EV–associated protein markers by western blotting analysis but has yet to be further validated by NTA or TEM. Refinement to PFEV isolation experimental protocols will be necessary for a comprehensive comparison of EV isolation techniques. Validation of the findings reported here would require further interrogation of prospectively collected thoracentesis samples.

Notwithstanding these limitations, this study demonstrates the feasibility of isolating the EV fraction of pleural fluid, and provides an understanding of the advantages and disadvantages of various methodologies for analysis of EV cargo in pleural effusion. We have shown that both microvesicles and exosomes can be isolated from archival pleural fluid samples, and that their miRNA cargo can be successfully profiled. This study lays groundwork and outlines a platform for future exploration of the extracellular vesicle compartment of pleural fluid to find novel diagnostic biomarkers of pleural disease.

## AUTHOR CONTRIBUTIONS

**T. M. Chee**: Conceptualization; data curation; formal analysis; funding acquisition; investigation; methodology; project administration; writing—original draft; writing—review and editing. **H. E. O'Farrell**: Methodology; writing—review and editing. **L. G. Lima**: Methodology; writing—review and editing. **A. Möller**: Conceptualization; funding acquisition; methodology; writing—review and editing. **K. M. Fong**: Conceptualization; supervision; writing—review and editing. **I. A. Yang**: Conceptualization; supervision; writing—review and editing. **R. V. Bowman**: Conceptualization; funding acquisition; supervision; writing—original draft; writing—review and editing.

## CONFLICTS OF INTEREST STATEMENT

The authors declare no conflicts of interest.
